# A novel in situ passive heating method for evaluating whole-tree responses to daytime warming in remote environments

**DOI:** 10.1186/s13007-022-00904-z

**Published:** 2022-06-11

**Authors:** Georgina A. Werkmeister, David Galbraith, Emma Docherty, Camilla Silva Borges, Jairo Matos da Rocha, Paulo Alves da Silva, Beatriz Schwantes Marimon, Ben Hur Marimon-Junior, Oliver L. Phillips, Emanuel Gloor

**Affiliations:** 1 School of Geography, University of Leeds, Leeds, UK; 2 Laboratório de Ecologia Vegetal, Campus de Nova Xavantina, Universidade do Estado de Mato Grosso, Nova Xavantina, Brazil

**Keywords:** Climate change impacts, Plant physiology, Passive heating, Open top chambers, Cerrado, In situ heating, Remote environments

## Abstract

**Background:**

Many significant ecosystems, including important non-forest woody ecosystems such as the Cerrado (Brazilian savannah), are under threat from climate change, yet our understanding of how increasing temperatures will impact native vegetation remains limited. Temperature manipulation experiments are important tools for investigating such impacts, but are often constrained by access to power supply and limited to low-stature species, juvenile individuals, or heating of target organs, perhaps not fully revealing how entire or mature individuals and ecosystems will react to higher temperatures.

**Results:**

We present a novel, modified open top chamber design for in situ passive heating of whole individuals up to 2.5 m tall (but easily expandable) in remote field environments with strong solar irradiance. We built multiple whole-tree heating structures (WTHSs) in an area of Cerrado around native woody species *Davilla elliptica* and *Erythroxylum suberosum* to test the design and its effects on air temperature and humidity, while also studying the physiological responses of *E. suberosum* to short-term heating. The WTHSs raised internal air temperature by approximately 2.5 °C above ambient during the daytime. This increased to 3.4 °C between 09:00 and 17:00 local time when thermal impact was greatest, and during which time mean internal temperatures corresponded closely with maximum ambient temperatures. Heating was consistent over time and across WTHSs of variable size and shape, and they had minimal effect on humidity. *E. suberosum* showed no detectable response of photosynthesis or respiration to short-term experimental heating, but some indication of acclimation to natural temperature changes.

**Conclusions:**

Our WTHSs produced a consistent and reproducible level of daytime heating in line with mid-range climate predictions for the Cerrado biome by the end of the century. The whole-tree in situ passive heating design is flexible, low-cost, simple to build using commonly available materials, and minimises negative impacts associated with passive chambers. It could be employed to investigate the high temperature responses of many understudied species in a range of complex non-forest environments with sufficient solar irradiance, providing new and important insights into the possible impacts of our changing climate.

**Supplementary Information:**

The online version contains supplementary material available at 10.1186/s13007-022-00904-z.

## Background

The Cerrado (Brazilian savannah), is the second largest biome in South America (originally covering approximately 2 million km^2^ [1]), is considered a ‘hotspot’ for biodiversity conservation [2] and is essential for maintaining major South America watersheds [3]. However, this important biome—like many significant non-forest woody ecosystems—has been historically undervalued and understudied [4, 5], and is experiencing the highest rates of land use change in Brazil [6]. It is also under immediate threat from climate change [7, 8], with regional climate models predicting a temperature increase of between 1 and 5 °C across the Cerrado biome by the end of this century [9]. Such increases in temperature have the potential to affect many aspects of plant growth and function [10], with repercussions for important plant–plant and plant–animal interactions [11, 12], making it essential to improve our understanding of high temperature responses of native vegetation. However, the majority of direct temperature manipulation experiments investigate low-stature species (such as tundra vegetation [13]), young saplings, or seedlings (for example [14, 15]) that can be grown in moveable units and climate-controlled chambers. Far fewer study adult individuals or larger, woody species, especially in situ, although such investigations would help provide more accurate predictions of how established communities and natural ecosystems will respond to climate change.

Targeted heating of organs, for example of leaves or branches [16–18], is a common method for studying temperature effects on larger individuals. However, this can lead to high variation in temperatures experienced by target organs [16], and may not realistically indicate the effects of climate warming, as temperature responses involve complex signals sometimes incorporating the whole plant [10, 19]. However, heating whole individuals typically requires greater inputs of time, equipment, and money, therefore few whole-tree in situ warming experiments have taken place (reviewed in [20]), with even fewer in the tropics [21, 22], likely due to the added difficulties of establishing experiments in often remote and harsh field environments.

In situ warming methods broadly fit into two categories; active, including using electrical heat resistance cables, infrared lamps, and active field chamber [20, 21]; and passive, including tents [23] and variations on open top chambers (OTCs) [24]. Although active methods can provide greater control over temperature and enable night-time warming [21], they can also overshoot target temperatures with detrimental effects (such as leaf-scorching [16]), and often require large amounts of electricity, necessitating access to AC power or a combination of portable power solutions, such as solar panels and batteries (as in [17]). Equipment can lead to high start-up costs, require electronics expertise, and be at risk of theft or malfunctioning over long-term experiments in remote and harsh environments. Conversely, passive warming methods that utilise solar radiation are often more appropriate in remote field sites where power access is often limited, and when portable power systems are impractical or prohibitively expensive.

OTCs, the most commonly employed method for passive warming [21], are particularly useful in long-term or smaller-budget studies, having low construction and maintenance costs. Traditionally OTCs are short and hexagonal in design, heating small areas of low-stature vegetation [24]. However, Chapman et al. [25], Welshofer et al. [26] and Coldren et al. [27] each employed modified OTCs to heat taller plant communities (up to 1.2, 1.5 and 2 m tall respectively) by approximately 1.8 °C during the daytime, and Miserere et al. [28] used 2 m cuboid OTCs to heat whole olive trees (transplanted in a cleared field) by approximately 3 °C during the daytime using solar heated rocks, although they also used electric fans. Chambers can also affect growing conditions by altering wind patterns; affecting internal gas concentrations, precipitation, light and humidity; and obstructing pollination and herbivory [21, 24]. However, OTCs traditionally have inclined sides or partially covered tops to aid heating, and uncovering the top completely (as in [25, 27]) can reduce such impacts.

To investigate how Cerrado shrubs and trees growing in situ (possibly already reaching their upper thermal limits [29]) will respond to daytime temperature increases they may experience by the end of this century, we developed a passive methodology for heating entire individuals of up to 2.5 m tall (larger than previously described methods and easily expandable) in situ by approximately 3 °C during the daytime, in line with mid-range climate predictions for the Cerrado [9]. Our novel in situ whole-tree passive heating structure (hereon referred to simply as whole-tree heating structure or WTHS) was designed to work in areas of low-canopy savannah and transition woodland (or similar areas with strong solar radiation), be low-cost and simple to build, use commonly available materials, and minimise the aforementioned artefacts of passive chambers.

The objective of this study was to test and demonstrate the functioning of our novel WTHS in the field. To further illustrate its usefulness we also carried out a short-term experiment investigating the photosynthesis and leaf dark respiration responses of Cerrado shrub *Erythroxylum suberosum* A.St.-Hil. (Erythroxylaceae) to in situ heating. Respiration typically increases exponentially with temperature, approximately doubling with every 10 °C, giving a Q_10_ (or proportional change in respiration rate given a 10 °C temperature increase) of approximately 2.0 [30]. Photosynthesis increases with temperature up to an optimum temperature (T_opt_) at which the rate of photosynthesis peaks (A_opt_) and then declines until zero at T_max_ [31]. Studies have suggested that tropical species are growing at temperatures close to their T_opt_ [32] and if temperatures increase above these values, photosynthesis may be reduced while respiration may continue to increase, leading to a reduction in the carbon available for growth, and possibly in the carbon storage potential of tropical ecosystems [18]. Acclimation of respiration (through a reduction in respiration rates at a given temperature or in Q_10_ [30]) or of photosynthesis (through an increase in T_opt_ or A_opt_ [32]) could help to reduce this imbalance, but there is limited evidence on whether tropical species, particularly in non-forest ecosystems, are capable of this acclimation over long or short timescales. Our study on *E. suberosum* demonstrates the possibilities of using our WTHS to gain insight into these and other important and complex temperature responses of understudied woody species in the Cerrado and similar remote but ecologically important non-forest ecosystems, such as the tapia woodlands of Madagascar and other neotropical savannahs [4].

## Materials and methods

### Field site

This study was conducted in the Bacaba Municipal Park (14° 42′ 28.8″ S, 52° 21′ 03.9 W), Nova Xavantina, Brazil, where the dominant physiognomy is typical Cerrado (Cerrado *sensu stricto*) [33] comprised of tree and shrub woodland, with a discontinuous upper layer of vegetation (tree cover 20–50%, average height 3–6 m [34]). The climate classification is Aw under the Köppen system, with a pronounced dry season (May–September), annual precipitation of 1300–1500 mm, and average monthly temperature of 25 °C according to the Nova Xavantina INMET (Brazilian Meteorological Service) climate station. In 2019, midday solar irradiance was 760 w/m^2^ on average, reaching up to 1170 w/m^2^, based on nearby WatchDog weather station data (Spectrum Technologies, Inc.).

### Structural design

#### Prototype whole-tree passive heating structure (WTHS)

**Fig. 1:**
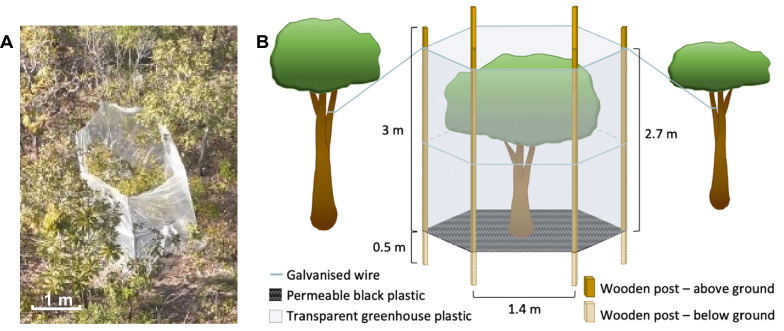
The novel, whole-tree in situ passive heating structure (WTHS). **A** Drone image of the prototype built in the Cerrado around an individual of *D. elliptica*. **B** Diagram depicting shape, size and materials

Our WTHS (Fig. 1) was developed to utilise solar radiation to passively heat whole individuals of woody species in semi-open environments like typical Cerrado. To test its functioning in a field situation, we built the prototype WTHS in a Cerrado location (Fig. 1A) around a 2.4 m tall (23.8 cm diameter at 30 cm) adult individual of *Davilla elliptica* A.St.-Hil. (Dilleniaceae), a dominant woody species both locally [33] and across much of the Cerrado biome [35] with ethnopharmacological uses [36]. The individual was chosen due to its average size within the local population, but otherwise at random.

**Fig. 2:**
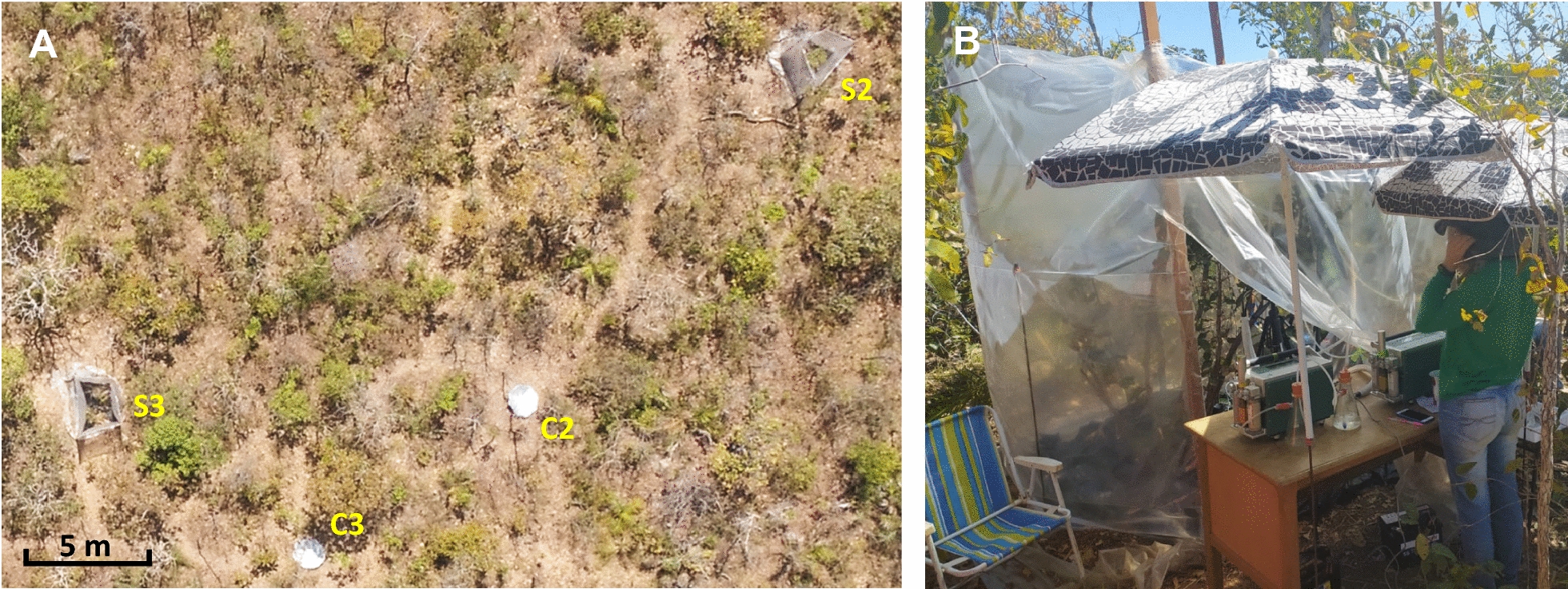
Four-sided WTHSs in situ in the Cerrado. **A** Aerial image of WTHSs S2 and S3 in situ in the Cerrado, with the position of control individuals C2 and C3 marked with umbrellas. **B** Photograph of WTHS S2 partially opened for taking measurements for the analysis of plant responses to temperature

The WTHS was designed with six sides (Fig. 1) similar to traditional OTCs [24]. Six 3.5 m long wooden posts (3 × 5 cm cross-section, with 30 mm U-shaped nails hit partially into them at heights of 1.8 and 3.4 m) were positioned 1.4 m apart around the chosen individual of *Davilla elliptica*, producing a hexagon with an overall perimeter of 8.4 m (Table 1) and diameter (from corner to corner) of 2.8 m. The posts were buried 50 cm deep, and galvanised steel wire was thread through the U-shaped nails at the top and middle of each post, joining the posts together to stabilise the structure and create the basic frame (Fig. 1B). Translucent white polythene film designed for greenhouses (4 m wide, 100 µm thick with UV protection, locally known simply as ‘plástico para estufa’) was held at 2.7 m (30 cm higher than the tree) and attached to each post in turn using 8 mm staples, creating the sides of the WTHS. The final edge of the plastic was attached in a temporary manner using wire, allowing easy detachment of the lower half, enabling the opening of a ‘door’ into the WTHS when required for testing (as seen on a later WTHS in Fig. 2B). Excess plastic at the bottom of the structure sides was covered with loose earth to seal the WTHS and prevent movement or tearing of the plastic on windy days. More wire was attached between the tops of the posts and nearby vegetation like guy ropes, providing extra stabilisation (Fig. 1B). Black polythene sheeting (as used in construction) was spread out around the tree, across the whole base of the WTHS (Fig. 1B), and holes were punched in it (taking care to avoid the root system) to allow movement of water to the soil underneath.

**Table 1:** Measurements for the prototype and three four-sided WTHSs (S1–S3)

Whole-tree heating structure	Length of side (cm)						Total perimeter length (cm)	Height (cm)	Cross-sectional area of base (m^2^)	Internal volume of WTHS (m^3^)
	1	2	3	4	5	6				
Prototype	140	140	140	140	140	140	840	270	5.09	13.74
S1	160	165	160	165	–	–	650	210	2.58	5.41
S2	130	135	255	200	–	–	720	280	2.93	8.19
S3	210	260	175	185	–	–	830	310	4.19	12.99

The black plastic base of the WTHS was designed to function as a passive heat source during sunny periods, absorbing incident solar radiation and emitting it as thermal radiation, while polythene film sides allowed high transmittance of shortwave, but low transmittance of longwave radiation, reducing thermal radiative loss [37]. The sides were built perpendicular to the ground and the top left completely open (as in [25, 27]), unlike in other OTC designs with inclined sides [24, 26], or partially covered tops [28]. This design aimed to maximise light transmission into the WTHS; avoid rain shadowing; and permit air movement between the inside and outside, preventing overheating and reducing the impact of the WTHS on gas exchange of the enclosed tree, the natural movement of pollinators or herbivores, and on microclimatic variables other than temperature (such as humidity); all of which are classic disadvantages of using chambers.

#### Experimental WTHSs

To further demonstrate the function and possible uses of our WTHS, we conducted a short-term experiment on the gas exchange acclimation response of *E. suberosum* (a woody Cerrado shrub) to in situ heating. For this we built three more WTHSs (S1, S2 and S3) in the Cerrado, each around an individual of *E. suberosum* (T1, T2 and T3; size data in Additional file 1: Fig. S3). These WTHSs were built using the prototype design, except with only four sides (Fig. 2), sized specifically to accommodate the smaller individuals (Table 1) or to encompass obstructing vegetation (WTHS S3). The plastic walls of each WTHS were erected at least 30 cm above the tallest branch of each individual.

### Abiotic measurement

Temperature and relative humidity (RH) data were recorded every minute using DTH22 sensors (Adafruit, NYC, USA; accurate to ± 0.5 °C and ± 2% RH) housed inside solar radiation shields (Windspeed Limited, Rhyl, UK), controlled by Arduino UNO (www.arduino.cc). To determine the impact of a WTHS on the internal microclimate, one sensor was placed at the centre of the WTHS, just above the crown of the enclosed individual (to avoid shading by the plant), while ambient temperature and RH were measured by a sensor placed above a nearby, similarly sized individual of the same species (acting as a control). Ambient air temperature, RH and solar irradiance were also measured every 15 min by the nearby WatchDog weather station.

Temperature and RH measurements for the prototype WTHS and nearby control individual of *D. elliptica* were taken continuously from June 9th to 27th 2020. Measurements taken inside the three four-sided WTHSs (S1–S3) built around individuals of *E. suberosum* (T1–T3) were compared with ambient readings taken at three corresponding control individuals (C1–C3). One pair of individuals was monitored at a time and the sensors were rotated between the three WTHSs (and their corresponding controls) over 3 weeks from August 4th 2020 (during the heating experiment). Climate data collected while WTHSs were open (for testing) were removed before analysis.

### Analysis of microclimate data

Temperature and RH measurements were averaged for every 15 min. Absolute water vapour content of the air (absolute humidity; AbsH) and vapour pressure deficit (VPD) were calculated from the temperature and RH data, according to the ideal gas law and Buck’s improved equation for saturation vapour pressure [38].

Mean diurnal patterns of internal and external temperature, RH and VPD were produced for each WTHS. Temperature and RH differences (between inside each WTHS and its corresponding control) were calculated for the daytime (06:30 to 18:30 local time), night-time (18:30 to 06:30), and between 09:00 and 17:00 each day (observed as the period of day with the strongest, most consistent levels of heating). We also compared temperature measurements with ambient temperatures recorded by the local weather station, and mean diurnal patterns of the relationships between them were calculated to enable estimation of temperatures for any WTHS on any day that direct measurement was not possible.

### Investigating plant responses to short-term in situ heating

To explore the suitability of our WTHS for direct field study, we conducted an in situ temperature manipulation experiment (July 27th to August 23rd 2020) to assess whether photosynthesis and dark respiration of *E. suberosum* could acclimate to the higher daytime temperatures reached inside the WTHS on a short time scale. *E. suberosum* is a woody shrub species common to the study area [33] and many parts of the Cerrado [35]. It was chosen due to this prevalence, its importance (having multiple medicinal uses [39]), and because at the time of the study in the latter half of the dry season, the leaves of *D. elliptica* (the species enclosed in the prototype WTHS) were not healthy enough for studying metabolism. Six individuals of *E. suberosum* were chosen for the study based on their similar size of ~ 2 m tall and 3 cm DSH (diameter at stump height or 30 cm; Additional file 1: Fig. S3) and leaf health. Three of these were randomly selected to be heated (treatment individuals; T1, T2 and T3), and the other three were designated as controls (C1, C2 and C3).

On the first morning of the experiment, photosynthesis and respiration temperature response curves were measured for the first treatment individual (T1), before the first four-sided WTHS (S1) was constructed around it. On the second morning, the same measurements were made for the first control individual (C1). This pattern was repeated for the remaining four trees (T2, C2, T3 and C3) and two WTHSs (S2 and S3). Each individual was re-measured 7, 14 and 21 days after the first measurements were made (and after heating began for the treatment individuals). The last set of respiration measurements (C3, day 21) was removed from the analysis as wind caused the leaf to rip during measurement. Physiological measurement methodology and analysis are described in Additional file 1: Appendix S1.

## Results

### Impact of WTHS on microclimate

**Fig. 3:**
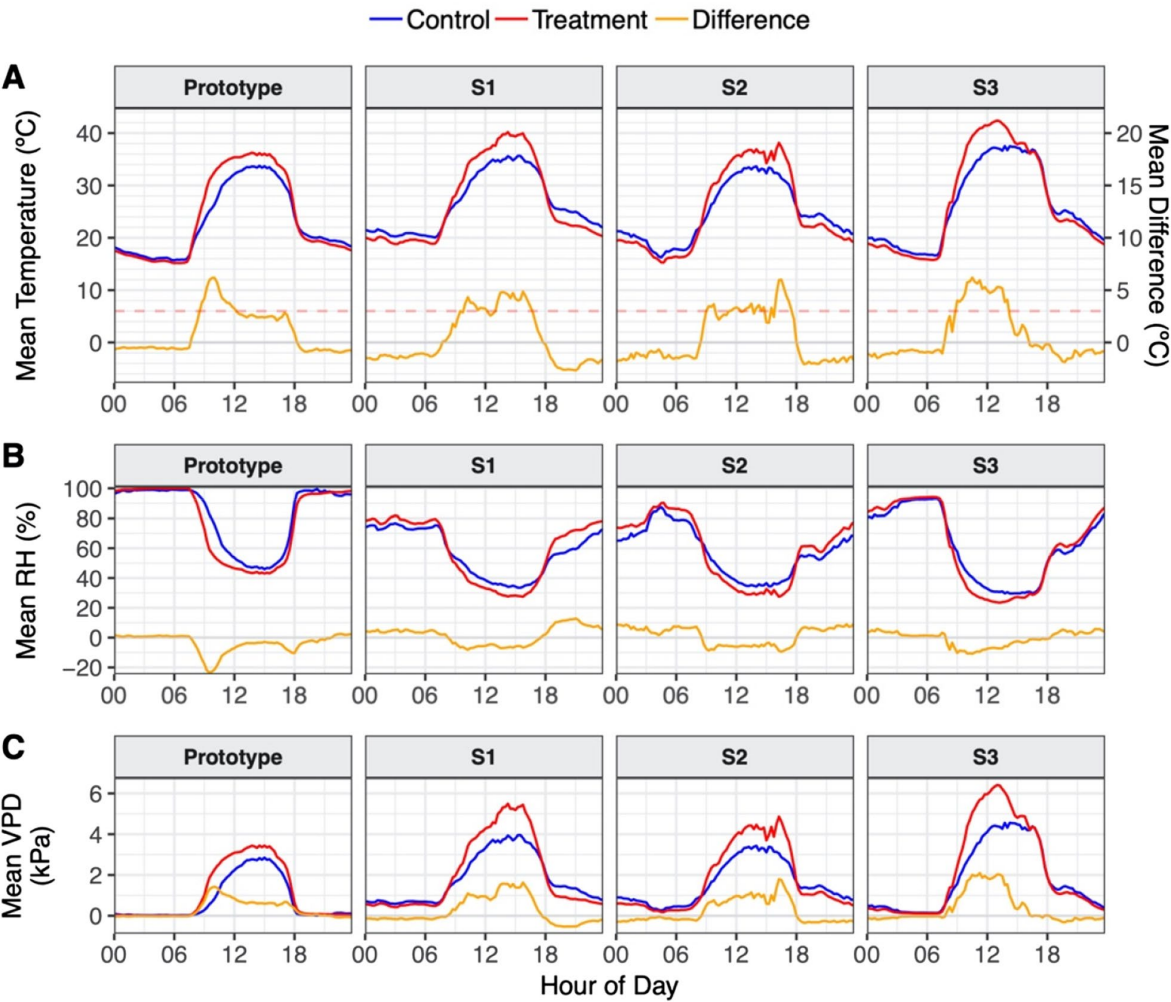
Mean diurnal patterns of **A** temperature; **B** relative humidity (RH); and **C** vapour pressure deficit (VPD); calculated from data recorded at treatment individuals inside each of the four WTHSs (the prototype, S1, S2 and S3; red lines), and their relative controls (blue lines), with the differences calculated between the two (orange lines). In **A** temperature difference is given on the right-hand axis for greater definition, and the dashed line indicates the target of 3 °C

**Fig. 4:**
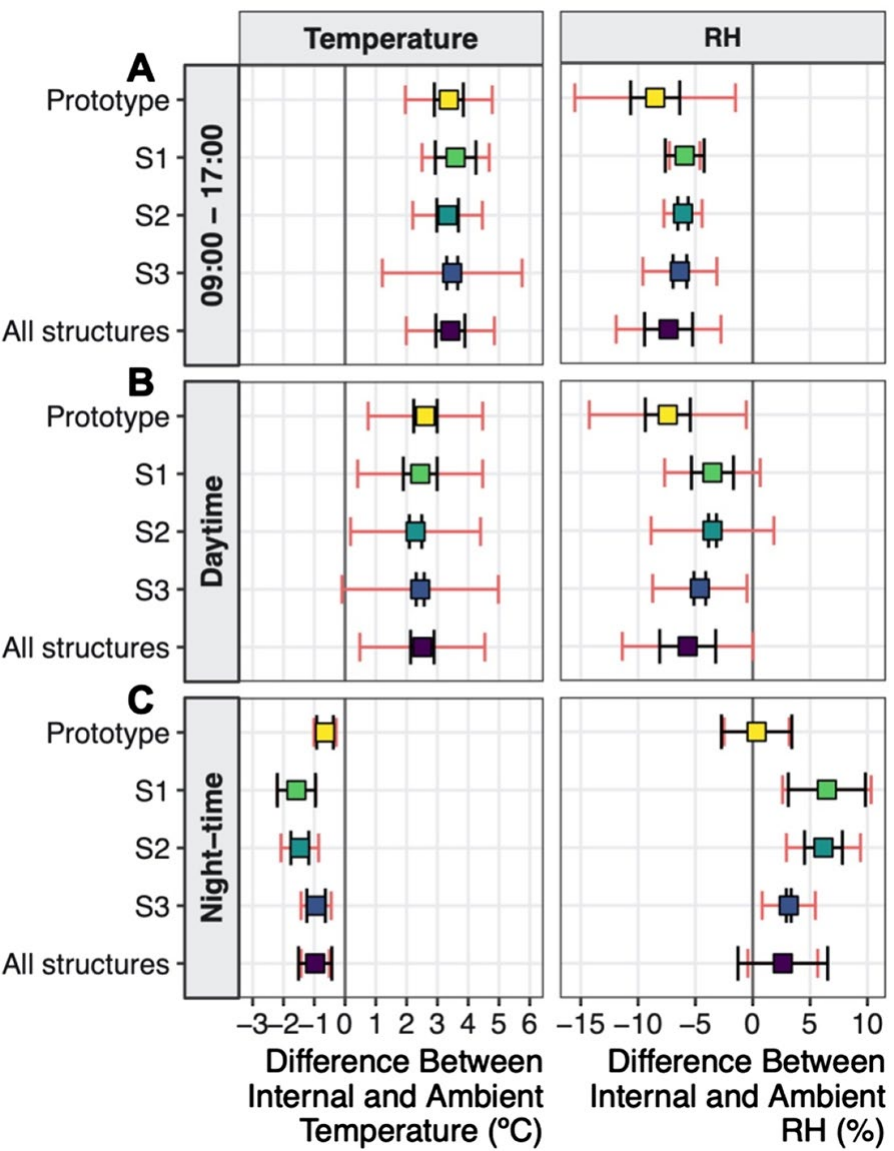
Mean differences in temperature and RH between inside each WTHS (prototype, S1, S2, S3) and their relative controls during given time periods, including average values for all four WTHSs together. Mean differences were calculated for **A** 09:00 to 17:00 (period of strongest heating), **B** daytime (06:30 to 18:30), and **C** night-time (18:30 to 06:30). Red error bars are the average (over all days of measurement) of the daily standard deviation of the mean difference in temperature or RH. Black error bars are the standard deviation of the daily mean differences over all days of measurement

Internal air temperature inside each WTHS correlated closely with ambient temperature, but rose higher in the daytime with increased irradiance (Fig. 3A). The mean difference between internal and ambient temperature (heating effect) during the daytime was 2.51 °C (± 2.03 °C SD) averaged across all four WTHSs, with little variation from day to day (SD between days of ± 0.38 °C) or structure to structure (Fig. 4B), although the strength of the heating effect varied between dawn and dusk. Each WTHS consistently produced and maintained a heating effect close to our target of 3 °C between 09:00 and 17:00 (Fig. 3A), averaging 3.42 °C across all WTHSs (± 1.43 °C SD; Fig. 4A). Again this varied little from day to day (SD between days of ± 0.47 °C) or structure to structure (Fig. 4A), except in WTHS S3 where heating fell around 14:00 (Fig. 3A), likely due to localised shading.

Maximum temperature differences generated by the WTHSs each day ranged between 4.80 and 7.66 °C depending on the day and the structure (Additional file 1: Fig. S4). The relationship between the minimum, mean and maximum temperatures recorded at the control individuals (between 09:00 and 17:00) was replicated very effectively within the WTHSs, approximately 3 °C higher, and mean internal temperatures repeatedly corresponded with maximum external temperatures (Additional file 1: Fig. S4).

There was no heating effect at night (between 18:30 and 06:30; Fig. 4C), and temperatures were on average 0.97 °C (± 0.46 °C) lower inside the WTHS than outside. This cooling effect was smaller in WTHS S3 than in the other four-sided WTHSs, and smallest in the prototype (Fig. 4C), the largest WTHS (Table 1).

The diurnal patterns of RH reflected those of temperature, showing lower RH inside each WTHS than outside during the daytime (Fig. 3B), with the biggest differences generated between 09:00 and 17:00 (when heating was strongest) when internal RH was on average 7.34% (± 4.57%) lower than ambient (Fig. 4A). At night, internal RH of all four-sided WTHSs was higher than ambient RH (Fig. 4C), particularly in S1 and S2 (6.46 and 6.16% higher on average respectively). High RH readings both inside and outside the prototype (Fig. 3B) meant no difference could be detected. Diurnal patterns of VPD corresponded with those of temperature, showing larger increases in internal VPD than ambient when heating was strongest (Fig. 3C). However, AbsH inside each WTHS remained close to ambient AbsH throughout the experiment (Additional file 1: Fig. S5).

### Response of *E. suberosum* to short-term heating

**Fig. 5:**
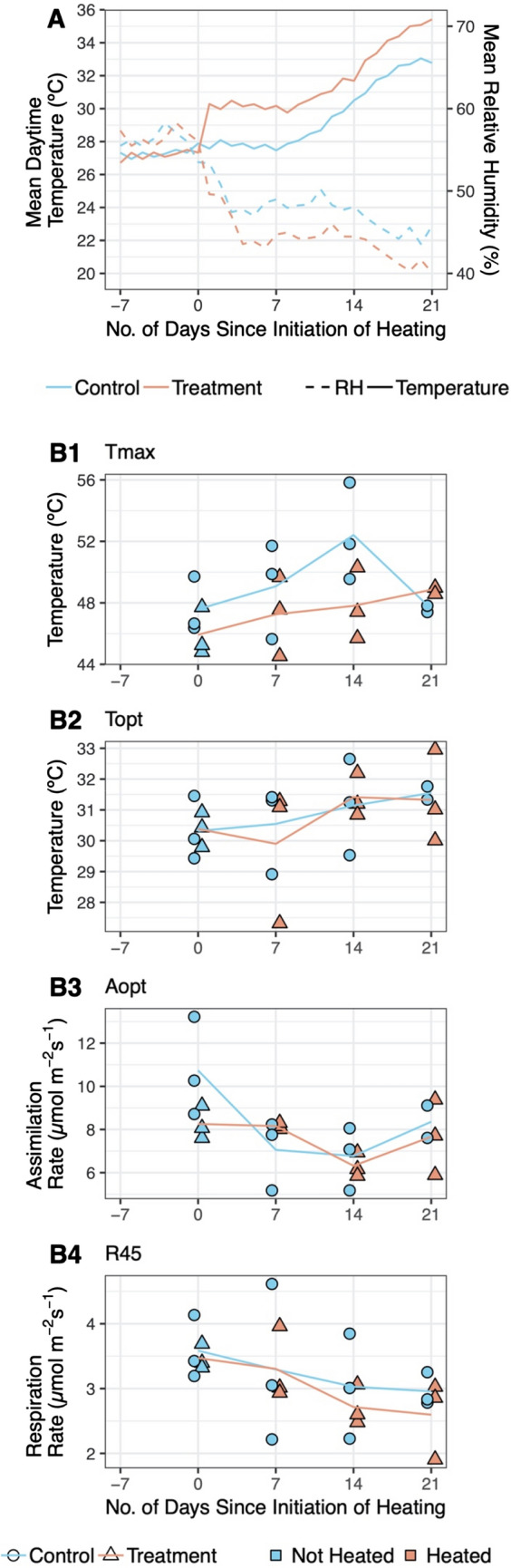
Climatic conditions and example results from the heating experiment on *E. suberosum*. **A** Mean daytime (06:30–18:30) temperature and RH experienced by treatment and control groups in relation to the number of days before or after heating began. **B1**–**B4** Results of the photosynthesis and respiration parameters **B1** T_max_, **B2** T_opt_, **B3** A_opt_, and **B4** R_45_, estimated from leaf measurements taken at 0, 7, 14 and 21 days after experiment initiation for each individual. Each point represents the results from one leaf on one day. Lines show average results for treatment and control groups

From the initiation of heating of *E. suberosum*, the treatment individuals experienced mean daytime temperatures 2.35 °C (± 2.13 °C SD) above the controls (Fig. 5A), increasing to 3.45 °C (± 1.44 °C) above between 09:00 and 17:00 (when the heating effect was strongest and most consistent). The RH was 3.81% lower on average during the daytime (± 4.37%; Fig. 5A) inside the treatment WTHSs than outside, and this difference increased to 6.05% (± 2.09%) between 09:00 and 17:00. Over the course of the experiment (day 0–21) there was also a large increase in mean ambient daytime temperature of 6.74 °C (Fig. 5A).

T-tests comparing the photosynthesis and respiration parameter results (T_max_, T_opt_, T_span_, A_opt_, R_25_, R_35,_ R_45_ and Q_10_) of each leaf showed no significant differences (*p* < 0.05) between the treatment and control groups either before or after the initiation of heating. Over the course of the experiment however, maximum and optimum temperatures for photosynthesis (T_max_ and T_opt_) showed increasing trends for both treatment and control groups (Fig. 5B1, B2), while respiration rates at given temperatures appeared to decrease (for example R_45_; Fig. 5B4) as did Q_10_ values for all individuals (except T2; Additional file 1: Fig. S6). Optimum photosynthesis rates (A_opt_) showed no obvious trend (Fig. 5B3).

Pearson correlation analysis of all results together showed no significant relationships (*p* < 0.05) between the photosynthesis parameter results of each leaf and temperatures experienced during the day or night prior to sampling. R_25_, R_35_ and R_45_ results each showed a significant negative relationship with the mean temperature of the previous day (*R* = − 0.45, *p* = 0.027; *R* = − 0.55, *p* = 0.005; *R* = − 0.45, *p* = 0.027; for R_25_, R_35_ and R_45_ respectively).

## Discussion

### Impact of WTHS on microclimate

The results demonstrate that our novel whole-tree in situ passive heating structure (WTHS) provides a useful and flexible methodology for direct experimental warming in order to investigate the effects of increasing daytime temperatures on woody plant species in semi-open field environments with strong solar irradiance. Air temperatures inside the WTHSs that we built and tested in the Brazilian Cerrado varied in accordance with ambient temperatures while producing a strong and reproducible heating effect of approximately 3.4 °C above ambient air temperature when irradiance was high (between 09:00 and 17:00 local time), a temperature increase that could be experienced across the Cerrado by 2100 given moderate emissions scenarios [9]. Furthermore, mean air temperatures inside the WTHSs closely matched maximum ambient temperatures, meaning the WTHSs exposed treatment individuals to realistic temperatures for the area, which they were already experiencing at a lower frequency.

While the maximum temperature differences between inside and outside the WTHSs sometimes reached as high as 7.66 °C, this often resulted from a delay in heating of the ambient environment (seen in the morning in the prototype; Fig. 3A), and the difference fell to approximately 3 °C once both internal and external environments reached their maximum daytime temperatures. Moreover, similar and sometimes more extreme spikes in temperature can also occur when employing active heating methods due to delays between the sensing of target temperatures and the deactivation of heating apparatus [16].

Night-time air temperatures were slightly lower inside the WTHSs than outside (Fig. 4C), however the inability to heat at night—sometimes accompanied by nocturnal cooling—is an unavoidable consequence of solar-powered passive heating methods [24]. The extent of cooling was lower in the larger WTHSs (the prototype and S3; Table 1), suggesting larger structures (with larger openings) are favourable as they allow more mixing of internal (cooler) and external (warmer) air, even during still nights. To increase night-time temperatures in their passive OTCs in Australia, Godfree et al. [40] used water filled plastic pipes to increase their thermal mass, and rocks could be used in a similar way. Although this method would likely be feasible in the Cerrado due to the high levels of solar irradiance, this addition could have reduced the heating effect produced by our WTHSs during the daytime [40] and we wanted to retain as strong and long a period of daytime heating as possible. However, this addition could be tested in the future, or by those wishing to employ our WTHS in their own research. More reliable night-time heating would require active methods, which are often expensive, technically complicated, and impractical in remote field sites such as the Cerrado. Conversely, our WTHS was designed to be entirely passive in order to provide a low-cost, easy to scale up methodology that does not require advanced technical knowledge or costly, high-tech equipment to set up.

As stated in the methods, the straight sides and entirely open top of our WTHS were designed to minimise common undesirable impacts of chambers, such as changes to microclimate variables other than temperature, in particular humidity. Nonetheless, RH still decreased and VPD increased inside each WTHS during the daytime, particularly during peak heating. However, AbsH remained largely unaffected by the WTHSs, suggesting that changes in RH and VPD were driven by changes in temperature and are an unavoidable consequence of temperature manipulation. Furthermore, RH has decreased over the Cerrado in recent years and could continue to fall with increasing temperatures [7], meaning our WTHSs may simulate more realistic climate change scenarios.

### Structural build and materials

The WTHS that we introduced here is simple to build and flexible in design. It can be modified to work with the locally available materials and equipment, as well as shaped to fit complex environments and different study species. Our initial, hexagonal design is appropriate for heating larger shrubs and trees as it can be efficiently expanded to encircle larger crown sizes. However, to surround *E. suberosum*—a small shrub species with a narrow growth form—it was more practical to erect four-sided WTHSs, saving materials, time and physical effort. For larger trees, if WTHSs need to be enlarged so there is more than 2 m between each post, more posts should be added to increase stability and reduce the likelihood of the plastic sides tearing in high winds (often experienced in the Cerrado).

Wooden posts were used to build the WTHS’s frame for strength and stability, even in high winds. Square cross-section metal posts (1.5 × 1.5 cm) were initially tested because they were lightweight and easier to transport, but they didn’t provide enough strength. However, if wood was not readily available, more robust metal posts could be a viable substitute, but would require testing first to ascertain their effect on internal temperature. We also plan to trial using locally sourced bamboo (an invasive species on nearby farms) in place of sawn wooden posts to reduce the cost and improve sustainability of the design. Using wood also allowed the use of U-shaped nails, which made attaching the wire frame easy and secure. Alternatives to wire for connecting the posts and securing the frame include various non-stretch cords (such as polyester or Kevlar rope), or wood or plastic poles for longer-term stability. Also, in areas where very hard ground impedes digging deep holes for the posts, reinforcing bar (rebar) can be hammered into the ground and fastened to the posts for additional support.

Since our WTHSs were built during the pronounced Cerrado dry season, permeability to water was not a principal concern. However, for use in wetter seasons or regions, the plain black polythene can be replaced with permeable alternatives to ensure the full movement of water, provided they remain black. Woven black polypropylene (designed for suppressing weeds in agricultural settings) is a low-cost and permeable alternative readily available in Brazil—known as ‘ráfia de solo’—and often available in regions where agriculture is common, which we plan to test in the future.

### Response of *E. suberosum* to short-term heating

The in situ heating of three individuals of *E. suberosum* provided no conclusive evidence of the higher daytime temperatures impacting photosynthesis, or of photosynthetic acclimation to short-term experimental heating, as no significant differences were found between the heated and control individuals for any of the parameters measured. It is possible that the period over which the individuals were heated (3 weeks) was too short to develop any detectable response, although photosynthetic acclimation has previously been observed after only 1 week of heating [41]. The sample size may also have been too small (three leaves tested per group per week) to detect any significant differences. In addition, ambient air temperature increased dramatically over the course of the experiment (Fig. 5A), which meant that in the latter half of the experiment, control individuals were experiencing temperatures as high as those produced inside the WTHSs roughly a week before. These high ambient temperatures could suggest that both the treatment and control individuals were already acclimated prior to the experiment to the higher temperatures produced. Interestingly, there was an increasing trend in T_max_ and T_opt_ (Fig. 5B1, B2) for both treatment and control groups which, although not statistically significant, suggests a possible acclimation to natural seasonal warming instead [42]. To investigate this further our experiment could be expanded to study a greater number of individuals—and species—for a longer duration.

Although we found no significant effects of short-term experimental heating on respiration rates when comparing the treatment individuals to the controls, Reich et al. [43] have shown that respiration rates may be more strongly controlled by night-time temperatures than daytime, and it is possible that stronger acclimation (upregulation of respiration) to the slightly cooler night-time temperatures experienced by the treatment individuals negated any acclimation (downregulation of respiration) to the higher daytime temperatures. Interestingly, the decreasing trends in both the estimated respiration rates of both groups (e.g. Fig. 5B4) and the Q_10_ of most individuals (Additional file 1: Fig. S6) throughout the experiment, along with the significant negative relationships found between the estimated respiration rates of all individuals and the temperatures experienced the day prior to taking measurements, suggest a possible downregulation of respiration in both groups and acclimation to the naturally increasing ambient temperatures [18]. However, deterioration in leaf health could also have contributed to these results as measurements were made towards the end of the growing season. Again, the results suggest the need for a longer and larger scale investigation in order to draw any reliable conclusions, which given more time would be achievable using our WTHS due to its low cost and versatile shape. Nevertheless, this study provides a clear demonstration of how our WTHS can be utilised in a range of in situ temperature manipulation experiments.

## Conclusions

Overall, the consistent level of heating produced by our novel, whole-tree in situ passive heating structure (WTHS)—regardless of size, shape, or enclosed species—demonstrates that the design is flexible and can be adapted for different individuals and field situations, while still providing a reproducible daytime heating effect in line with mid-range climate predictions for the Cerrado biome by the end of this century. Production of a strong heating effect without the more traditional inclined walls or reduced openings decreases the likelihood of non-desirable chamber effects, and the lack of any electricity requirement means that the design can be widely used in long-term investigations, provided solar irradiance is sufficient. Low-cost and simple to build, our WTHS can be easily scaled up to apply one treatment scenario to multiple taller-stature individuals in situ (as we did with *E. suberosum*), particularly in complex environments that don’t allow for construction of uniform chambers, providing new opportunities to gain vital insight into the high temperature responses of understudied shrub and tree species in remote environments.

## Supplementary Information

**Additional file 1: Appendix S1.** Investigating plant responses to short-term in situ heating, additional methods. **Appendix S2.** Additional figures; **Figure S3.** Size data of *E. suberosum* individuals. **Figure S4.** Daily temperatures in WTHSs. **Figure S5.** Absolute humidity results for WTHSs. **Figure S6.** Q_10_ results of *E. subersoum* individuals.

## Data Availability

Data will be archived to the NERC EIDC upon publication.
