# Supplementary material for: A novel in situ passive heating method for evaluating whole-tree responses to daytime warming in remote environments
Source: Plant Methods. 2022 Jun 11;18:78. doi: 10.1186/s13007-022-00904-z (PMC9188097; doi:10.1186/s13007-022-00904-z)
Supplement: Supplementary file 1 — Additional file 1: Appendix S1. Investigating plant responses to short-term in situ heating, additional methods. Appendix S2. Additional figures; Figure S3. Size data of E. suberosum individuals. Figure S4. Daily temperatures in WTHSs. Figure S5. Absolute humidity results for WTHSs. Figure S6. Q10 results of E. subersoum individuals. [file 13007_2022_904_MOESM1_ESM.docx]

**ADDITIONAL FILE 1**

**A novel *in situ* passive heating method for evaluating whole-tree responses to daytime warming in remote environments**

Georgina A. Werkmeister*, David Galbraith, Emma Docherty, Camilla Silva Borges, Jairo Matos da Rocha, Paulo Alves da Silva, Beatriz Schwantes Marimon, Ben Hur Marimon-Junior, Oliver L. Phillips, Emanuel Gloor.

*Email: [gygaw@leeds.ac.uk](about:blank)

**TABLE OF CONTENTS**

[Appendix S1: Investigating plant responses to short-term *in situ* heating, additional methods 2](#_Toc95988121)

[1.1 Physiological measurements 2](#_Toc95988122)

[1.2 Analysis of physiological response to temperature 3](#_Toc95988123)

[1.3 References 4](#_Toc95988124)

[Appendix S2: Additional figures 5](#_Toc95988125)

Appendix 1: Investigating plant responses to short-term *in situ* heating, additional methods

1.1 Physiological measurements

Photosynthesis and respiration temperature response curves were measured between 08:00 and 11:30 each day using two LI-6400XT portable photosynthesis systems (LI-COR Biosciences Inc., Nebraska, USA), one with a fluorometer (6400-40) chamber head and one with an LED (6400-02B) chamber head (for photosynthesis and respiration measurements respectively). One leaf was analysed with each LI-6400XT (two leaves per individual per day). Leaves were chosen that were healthy, fully expanded and equally mature, avoiding damage as much as possible. Leaves chosen for respiration analysis were covered at dawn to prevent sun exposure and kept in darkness for at least 40 minutes before testing began, while those for photosynthesis analysis were exposed to direct sunlight for at least 40 minutes prior to testing. Once the LI-6400XT chamber heads were clamped onto the still attached leaves (Figure S1), leaf temperature and either net photosynthesis or respiration rates were measured at 10 incremental steps in air temperature between 20 and 50°C, after full equilibration at each temperature. Temperatures above 40°C were achieved using temperature expansion water jackets (LI-COR Biosciences Inc.). Throughout measurement, CO_2_ concentration was maintained at 400 ppm and relative humidity (RH) at 50% for all leaves, while light levels were maintained at 1100 µmol m^-2^ s^-1^ for photosynthesis leaves and at 0 µmol m^-2^ s^-1^ for respiration leaves.


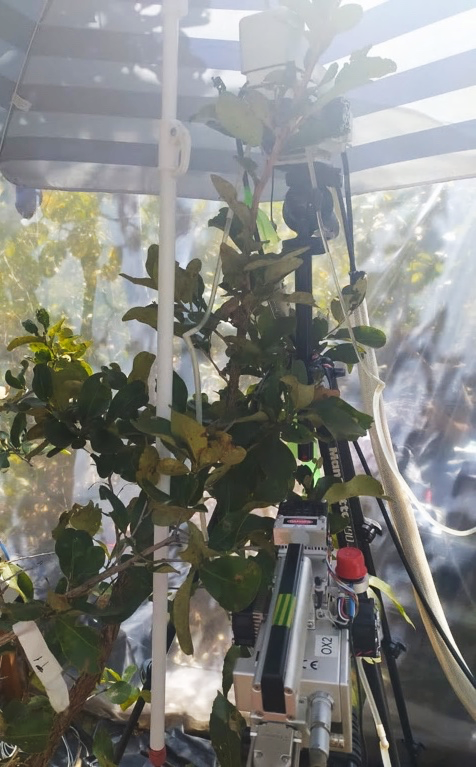


**Figure S1.** Image of both LI-6400XT chamber heads clamped onto leaves of a treatment individual of *E. suberosum* while taking photosynthesis and respiration measurements inside one of the WTHSs (whole tree heating structures). The chamber heads were mounted on tripods to enable them to reach the leaves whilst still attached, and shaded by umbrellas to control temperature.

1.2 Analysis of physiological response to temperature

Net photosynthesis rates (A_net_) were plotted against leaf temperature for each individual on each day of sampling, and a standard quadratic curve was fit to each one (Figure S2A). As the data fit to this equation was good for most leaves (R^2^ > 0.67, mean R^2^ = 0.88) no further models were tested. From the curves the optimum photosynthesis rate (A_opt_), optimum temperature for photosynthesis (T_opt_), maximum temperature for photosynthesis (T_max_), and temperature range at which photosynthesis is at least 80% of A_opt_ (T_span_) were extracted.


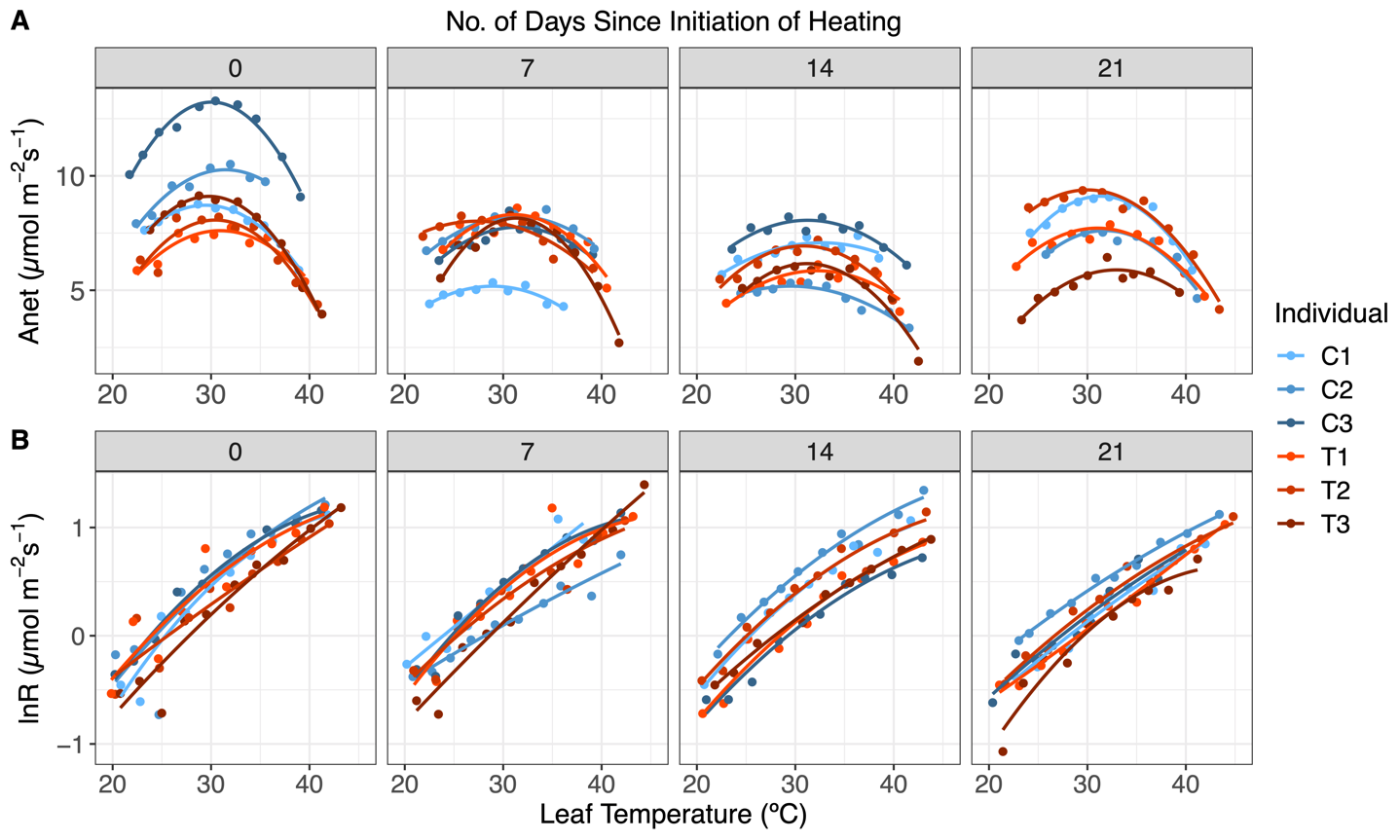


**Figure S2.** Measured values and fitted temperature response curves of (A) net photosynthesis rate and (B) respiration rate (natural-log-transformed) for each leaf on each day of measurement.

Respiration rates (R) were natural-log-transformed (*ln*) and plotted against leaf temperature for each individual on each day of sampling, and a quadratic curve was fit to each one (similar to [1]; Figure S2B). The data fit to this equation was strong for most leaves (R^2^ > 0.80, mean R^2^ = 0.94) so no further models were tested. From these curves, values were then extracted for respiration rates at low (25°C; R_25_), moderate (35°C; R_35_), and high temperatures (45°C; R_45_) for each individual and each day of measurement. Respiration rates R_20_ and R_40_ (at 20 and 40°C; a range of temperatures often experienced by *E. suberosum* individuals in the area) were also taken from the curves and used to calculate a Q_10_ value for the respiration rate of each individual on each day of measurement (using eq. 1 in [2]).

To evaluate whether the higher daytime temperatures experienced by the treatment individuals of *E. suberosum* had an impact on the temperature sensitivity of photosynthesis and respiration, all the parameters (namely T_max_, T_opt_, T_span_, A_opt_, R_25_, R_35,_ R_45_ and Q_10_; described above) calculated from measurements taken in the 3 weeks after the initiation of heating were compared between the treatment and control groups using 2-sided t-tests. The first two weeks of results post-heating were also tested separately in case a deterioration in general leaf health (which had been noticed in the field) was having an impact on the results by the third week of analysis. T-tests were also used to compare all the parameter results between the groups prior to heating to check for significant differences between the treatment and control groups that may have existed before the experiment began.

To further explore whether air temperature affected the temperature sensitivity of photosynthesis and respiration regardless of grouping, mean daytime and night-time temperatures were calculated for every 24 hours and every *E. suberosum* individual, and Pearson correlation analysis carried out between all photosynthesis and respiration parameters and the mean daytime and night-time temperatures of the day before leaf measurements were taken. Missing temperature data was estimated from temperature data recorded by the local weather station, and the previously calculated diurnal patterns of temperature differentials (see main article methods). The temperatures recorded on the days of testing were not used because leaf analysis started so early in the day that the leaves were not exposed to these temperatures before manipulation by the LI-COR systems. All data analysis was performed in R version 3.5.3 [3].

1.3 References

1. Heskel MA, O’Sullivan OS, Reich PB, Tjoelker MG, Weerasinghe LK, Penillard A, et al. Convergence in the temperature response of leaf respiration across biomes and plant functional types. Proc Natl Acad Sci. 2016;113:3832–7.

2. Atkin OK, Tjoelker MG. Thermal acclimation and the dynamic response of plant respiration to temperature. Trends Plant Sci. 2003;8:343–51.

3. R Core Team. R: A language and environment for statistical computing [Internet]. R Foundation for Statistical Computing, Vienna, Austria; 2019. Available from: https://www.R-project.org/

Appendix 2: Additional figures

**Figure S3.** Height and diameter at stump height (DSH; 30cm) of the three treatment individuals of *E. suberosum* (T1, T2, and T3) enclosed in the four-sided WTHSs (S1, S2 and S3 respectively); the individuals marked as controls (C1, C2 and C3); and the mean values for each group and all individuals together. Error bars denote standard deviation.


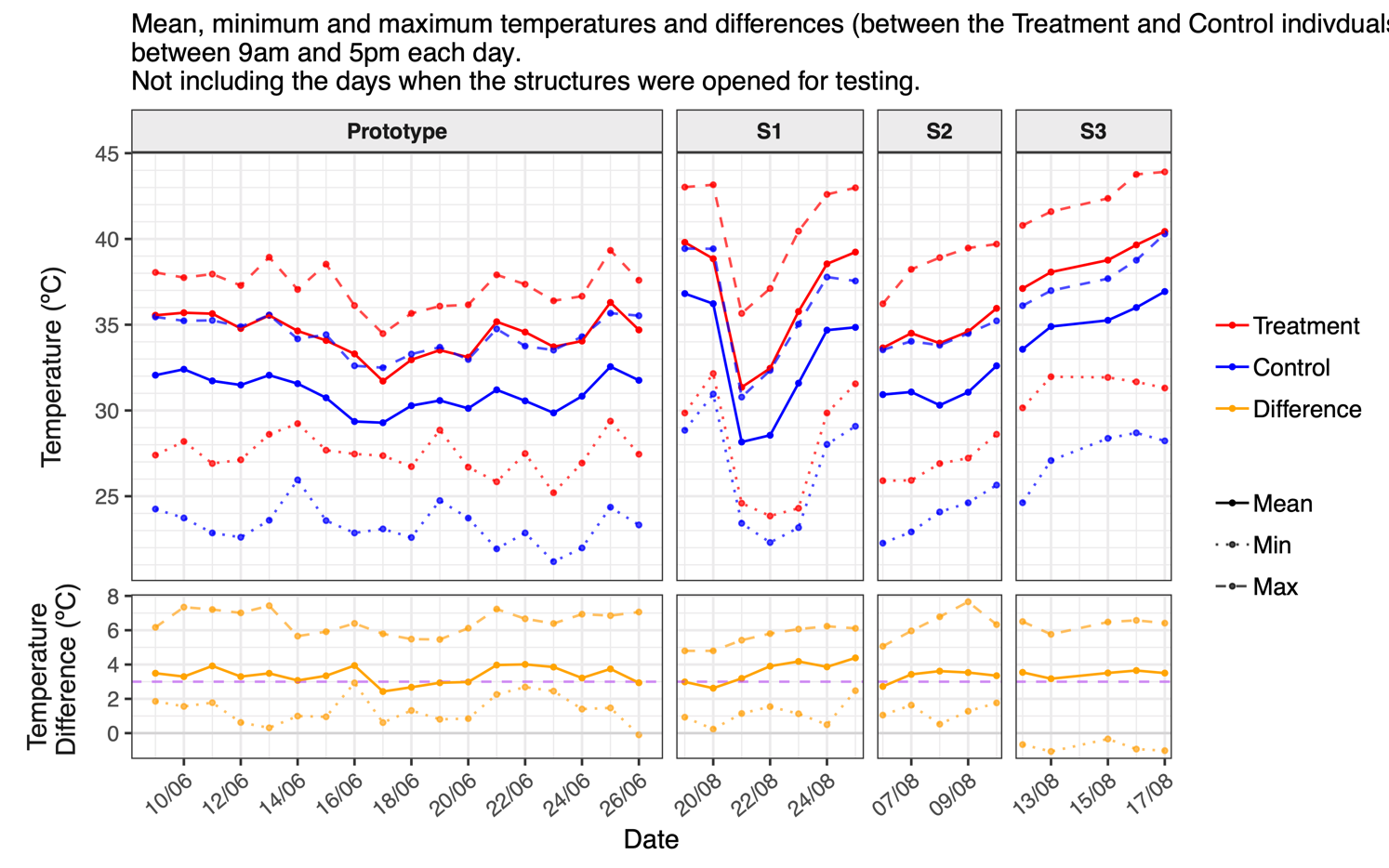


**Figure S4.** Mean, minimum and maximum temperatures experienced at the treatment individuals in each of the WTHSs, and at their respective control individuals during the period of strongest heating (between 09:00 and 17:00) on each day of temperature recording that WTHSs were left closed throughout; with the mean, minimum and maximum differences in temperature between inside and outside the WTHSs (during this period of each day) given in the lower panel. The purple dashed line indicates the target temperature difference of 3 °C.


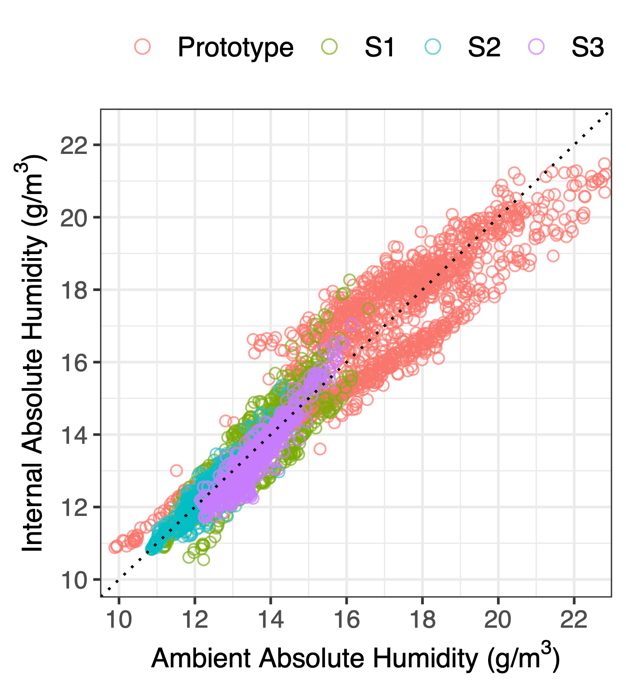


**Figure S5.** Absolute humidity compared between the air inside each of the WTHSs and the ambient air outside, at each of their corresponding control individuals. The dotted line represents a 1:1 relationship.


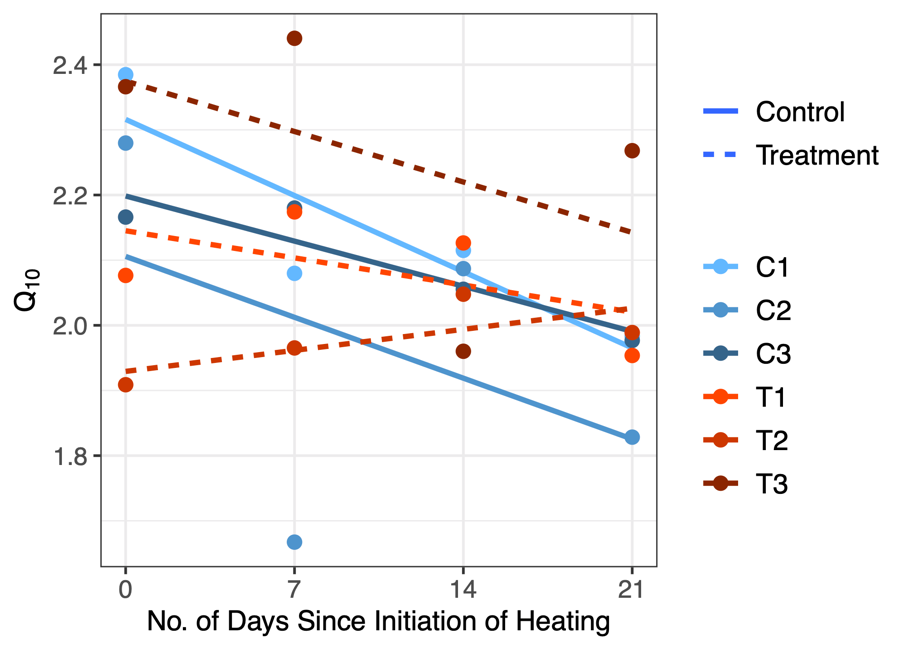


**Figure S6.** Q_10_ results calculated between 20 and 40°C from measurements taken from each tree on each day of sampling. The trendline for each tree is shown for clarity, but there were no significant trends found within a 95% confidence limit.
